# CEOP/IVE/GDP alternating regimen compared with CEOP as the first-line therapy for newly diagnosed patients with peripheral T cell lymphoma: results from a phase 2, multicenter, randomized, controlled clinical trial

**DOI:** 10.1186/s13073-020-00739-0

**Published:** 2020-04-30

**Authors:** Ming-Ci Cai, Shu Cheng, Xin Wang, Jian-Da Hu, Yong-Ping Song, Yao-Hui Huang, Zi-Xun Yan, Yu-Jie Jiang, Xiao-Sheng Fang, Xiao-Yun Zheng, Li-Hua Dong, Meng-Meng Ji, Li Wang, Peng-Peng Xu, Wei-Li Zhao

**Affiliations:** 1 State Key Laboratory of Medical Genomics, Shanghai Institute of Hematology, National Research Center for Translational Medicine, Shanghai Rui Jin Hospital, Shanghai Jiao Tong University School of Medicine, 197 Rui Jin Er Road, Shanghai, 200025 China; 2 Department of Hematology, Shandong Provincial Hospital Affiliated to Shandong University, Jinan, China; 3 Fujian Institute of Hematology, Fujian Medical University Union Hospital, Fuzhou, China; 4 Department of Hematology, The Affiliated Cancer Hospital of Zhengzhou University, The First Affiliated Hospital of Zhengzhou University, Zhengzhou, China; 5 Pôle de Recherches Sino-Français en Science du Vivant et Génomique, Laboratory of Molecular Pathology, Shanghai, China

**Keywords:** Peripheral T cell lymphoma, Alternating regimen, CHOP, Overall response rate, Prognosis, Prognostic biomarker

## Abstract

**Background:**

Cyclophosphamide, doxorubicin, vincristine, and prednisolone (CHOP)/CHOP-like chemotherapy is widely used in peripheral T cell lymphoma (PTCL). Here we conducted a phase 2, multicenter, randomized, controlled trial, comparing the efficacy and safety of CEOP/IVE/GDP alternating regimen with CEOP in newly diagnosed PTCL.

**Methods:**

PTCL patients, except for anaplastic large cell lymphoma-anaplastic lymphoma kinase positive, were 1:1 randomly assigned to receive CEOP/IVE/GDP (CEOP, cyclophosphamide 750 mg/m^2^, epirubicin 70 mg/m^2^, vincristine 1.4 mg/m^2^ [maximum 2 mg] on day 1, and prednisone 60 mg/m^2^ [maximum 100 mg] on days 1–5 every 21 days, at the first and fourth cycle; IVE, ifosfamide 2000 mg/m^2^ on days 1–3, epirubicin 70 mg/m^2^ on day 1, and etoposide 100 mg/m^2^ on days 1–3 every 21 days, at the second and fifth cycle; and GDP, gemcitabine 1000 mg/m^2^ on days 1 and 8, cisplatin 25 mg/m^2^ on days 1–3, and dexamethasone 40 mg on days 1–4 every 21 days, at the third and sixth cycle) and CEOP (every 21 days for 6 cycles). Analysis of efficacy and safety was of the intent-to-treatment population. The primary endpoint was a complete response rate at the end of treatment. Meanwhile, whole exome sequencing and targeted sequencing were performed in 62 patients with available tumor samples to explore prognostic biomarkers in this cohort as an exploratory post hoc analysis.

**Results:**

Among 106 patients, 53 each were enrolled to CEOP/IVE/GDP and CEOP. With 51 evaluable patients each in two groups, a complete response rate of the CEOP/IVE/GDP group was similar to that of the CEOP group (37.3% vs. 31.4%, *p* = 0.532). There was no difference in median progression-free survival (PFS; 15.4 months vs. 9.2 months, *p* = 0.122) or overall survival (OS; 24.3 months vs. 21.9 months, *p* = 0.178). Grade 3–4 hematological and non-hematological adverse events were comparable. Histone modification genes were most frequently mutated (25/62, 40.3%), namely *KMT2D*, *KMT2A*, *SETD2*, *EP300*, and *CREBBP*. Multivariate analysis indicated that *CREBBP* and *IDH2* mutations were independent factors predicting poor PFS and OS (all *p* < 0.001), while *KMT2D* predicting poor PFS (*p* = 0.002).

**Conclusions:**

CEOP/IVE/GDP alternating regimen showed no remission or survival advantage to standard chemotherapy. Future clinical trials should aim to develop alternative regimen targeting disease biology as demonstrated by recurrent mutations in epigenetic factors.

**Trial registration:**

The study was registered on ClinicalTrial.gov (NCT02533700) on August 27, 2015.

**supplementary material:**

**Supplementary information** accompanies this paper at 10.1186/s13073-020-00739-0.

## Background

Peripheral T cell lymphoma (PTCL) is a heterogeneous entity of non-Hodgkin lymphoma (NHL) with aggressive clinical behavior. Although cyclophosphamide, doxorubicin, vincristine, and prednisolone (CHOP) or CHOP-like chemotherapy is widely used in PTCL [1, 2], a complete response rate (CRR) ranges from 35.9 to 65.8% and the 5-year overall survival (OS) rate is 38.5% for all PTCL patients receiving front-line anthracycline-based treatment [3]. Multiple combinational regimens have been attempted in PTCL, including gemcitabine, cisplatin, prednisone (GDP) [4], and ifofamide, epirubicin, and etoposide (IVE) [5–10]. Furthermore, alternating regimen as CHOP/IVE/MTX showed promising anti-tumor activity by improving response rate in enteropathy-associated T cell lymphoma (EATL) [11]. However, randomized and controlled trials are still lacking in this field.

Dysregulated epigenetic mechanisms play a central role in the pathogenesis of PTCL. Main genes involved in epigenetic regulation are frequently mutated in PTCL, including histone modification (*KMT2D*, *KMT2A*, *SETD2*, *KDM6A*, *EP300*, and *CREBBP*), chromatin remodeler (*ARID1B* and *ARID2*) [12], and DNA methylation and demethylation genes (*TET2*, *DNMT3A*, and *IDH2*) [13]. We previously reported that histone modification gene mutations are associated with inferior progression-free survival (PFS) time of the patients [12]. However, the prognostic value of individual epigenetic regulator remains to be further investigated in PTCL, particularly for identifying patients with poor prognosis who may benefit from targeted agents.

Given the poor response to CHOP-based regimens and the potential anti-lymphoma activity by alternating chemotherapy in PTCL, we conducted a phase 2, multicenter, randomized, controlled trial, comparing the efficacy and safety of CEOP/IVE/GDP alternating regimen with CEOP in a Chinese cohort of newly diagnosed patients with PTCL. Meanwhile, we performed whole exome sequencing (WES) and targeted sequencing in 62 patients with available tumor samples to explore prognostic biomarkers in this prospective cohort. Results from this study were previously presented as an abstract [14].

## Methods

### Study design and patients

This phase 2, multicenter, randomized, controlled trial was conducted at four centers (Additional file 1: Table S1) in China within cooperative network of the Multicenter Hematology-Oncology Programs Evaluation System (M-HOPES). Eligible patients were ≥ 16 years of age with newly diagnosed, histologically confirmed PTCL according to 2008 WHO classifications [15]: peripheral T cell lymphoma-not otherwise specified (PTCL-NOS), angioimmunoblastic T cell lymphoma (AITL), anaplastic large cell lymphoma (ALCL)-anaplastic lymphoma kinase (ALK) negative, EATL, subcutaneous panniculitis like T cell lymphoma, and hepatosplenic T cell lymphoma (HSTL). Patients were required to have WHO performance status of ≤ 2, no previous history of malignancy, radiologically measurable disease, and a life expectancy of ≥ 6 months. Patients were not eligible once meeting any of the following criteria: NK/T cell lymphoma, ALCL-ALK positive, or primary central nervous system (CNS) lymphoma, previous systemic chemotherapy or local therapy, previous hematopoietic stem cell transplantation (HSCT), antifungal or antiviral therapy, uncontrollable cardiocerebrovascular, coagulative, autoimmune, or serious infectious disease, left ventricular ejection fraction (LVEF) ≤ 50%, other uncontrollable medical condition that may interfere with their participation in the study. Patients were also not enrolled if they had inadequate renal, hepatic, or bone marrow function, were not able to comply with the protocol for any reasons, were pregnant or breastfeeding, or had active liver or biliary disease, or human immunodeficiency virus (HIV) infection.

The study was approved by the Institutional Review Boards of all centers. Informed consent was obtained from all patients in accordance with the Declaration of Helsinki. The study was registered on ClinicalTrial.gov (NCT02533700) (https://www.clinicaltrials.gov/ct2/show/NCT02533700?term=NCT02533700&rank=1) on August 27, 2015. The trial was overseen by trial management and trial steering committees. The CONSORT checklist for reporting a randomized trial can be found in Additional file 2.

### Whole exome sequencing

Genomic DNA was extracted from frozen tumor samples of 27 patients [16, 17] and peripheral blood of 21 patients using Wizard® Genomic DNA Purification Kit (Promega, Wisconsin-Madison, USA) according to the manufacturer’s instructions. Exome regions of all samples were captured using a SeqCap EZ Human Exome kit (version 3.0) and sequenced on HiSeq 4000 platform with 150 bp paired-end strategy. Read pairs were then aligned to Refseq hg19 by Burrows-Wheeler Aligner (BWA) version 0.7.13-r1126, with PCR duplications removed by Samtools version 1.3 to generate chromosomal coordinate-sorted bam files. The mean depth of each sample was 90.1× (range 68.7–107.2×), with an average 98.2% (range 96.9~97.9%) of the target sequence being covered sufficiently deep for variant calling (≥ 10× coverage). A total of 33 genes were selected based on two criteria: (i) genes with recurrent mutations (> 3%) and/or (ii) genes with relevance to oncogenesis of PTCL (Additional file 1: Table S2). Population-related variants were eliminated using the variants in the 1000 Genomes Project, and SNP mutations were identified by COSMIC (the Catalogue Of Somatic Mutations In Cancer version 77).

### Targeted sequencing

Genomic DNA was extracted from frozen or paraffin tumor samples of 35 PTCL patients using a QIAamp DNA Mini Kit (Qiagen, Hilden, Germany) according to the manufacturer’s instructions. PCR primers were designed by Primer 5.0 software and listed in Additional file 1: Table S2. Multiplexed libraries of tagged amplicons from PTCL tumor samples were generated by Shanghai Yuanqi Bio-Pharmaceutical Multiplex-PCR Amplification System. Deep sequencing was performed using established Illumina protocols on HiSeq 4000 platform (Illumina).

### Study treatment

Patients in CEOP/IVE/GDP group received intravenous cyclophosphamide 750 mg/m^2^, epirubicin 70 mg/m^2^, vincristine 1.4 mg/m^2^ (maximum 2 mg) on day 1, and oral prednisone 60 mg/m^2^ (maximum 100 mg) on days 1–5 every 21 days, at the first and fourth cycle with CEOP. Intravenous ifosfamide 2000 mg/m^2^ on days 1–3, epirubicin 70 mg/m^2^ on day 1, and etoposide 100 mg/m^2^ on days 1–3 every 21 days, at the second and fifth cycle with IVE. Intravenous gemcitabine 1000 mg/m^2^ on days 1 and 8, cisplatin 25 mg/m^2^ on days 1–3, and dexamethasone 40 mg on days 1–4 every 21 days, at the third and sixth cycle with GDP, for a total of 6 cycles. Patients in the CEOP group received CEOP regimen every 21 days for 6 cycles.

Supportive care was provided according to institutional clinical practice. Granulocyte-colony stimulating factor (G-CSF) was administered if absolute neutrophil count was < 1.0 × 10^9^/L. Consolidation radiotherapy was permitted for patients with residual disease at EOT, if existed. Transplantation eligible patients were recommended to receive HSCT once complete response (CR) or partial response (PR) was achieved. Second-line treatment was considered if the patient had stable disease (SD) at interim evaluation. Prophylaxis for CNS relapse was given to patients with involvement of bone marrow, nasal/paranasal sinuses, orbit, breast, or testis.

### Randomization and masking

Patients were randomly assigned to two study groups by computer-assisted permuted-block (block size of 4, allocation ratio 1:1), receiving either CEOP/IVE/GDP alternating regimen or CEOP regimen. After obtaining informed consent from the patients, the investigators went online at the data center for registration and central randomization to assign treatment. A statistician located centrally in Shanghai Rui Jin Hospital supervised the randomization procedure. Investigators and patients were not masked to treatment assignment due to different ways of administration by each treatment group.

### Assessments

Positron emission tomography-computed tomography (PET-CT) or CT scans with contrast were evaluated at baseline, after 3 cycles for interim evaluation and at EOT for final evaluation. Treatment responses were assessed according to 2014 Lugano criteria for non-Hodgkin lymphoma [18]. Central response assessment of PET and CT images was performed by radiologists of Shanghai Ruijin Hospital, who was not informative of the treatment group. CT scans of the neck, thorax, abdomen, and pelvis were repeated every 3 months thereafter to monitor disease progression in the first year, then every 6 months in the following 2 years, and yearly thereafter. The severity of adverse events was assessed according to the Common Terminology Criteria for Adverse Events (CTCAE) version 4.0.

Baseline clinical laboratory tests and examinations included complete blood cell count (CBC), serum lactate dehydrogenase (LDH), hepatitis B virus DNA (HBV-DNA), HIV, coagulation function including APTT, PT and fibrinogen, bone marrow aspiration and trephine biopsy, electrocardiography, echocardiography, PET-CT, or CT scans with contrast. Vital signs should be recorded at each visit. International Prognostic Index (IPI), National Comprehensive Cancer Network IPI (NCCN-IPI), and Prognostic Index for T cell lymphoma (PIT) were calculated for each patient at baseline.

### Outcomes

The primary endpoint in this study was CRR at EOT, measured by PET-CT or CT scans with contrast, according to 2014 Lugano classification. The secondary endpoints included PFS (defined as the duration time between the date of randomization and the date of disease progression or death from any cause), OS (defined as the duration time between the date of randomization and the date of death from any cause), overall response rate (ORR) at EOT, and the toxicity, evaluated according to the National Cancer Institute Common Terminology Criteria of Adverse Events, version 4.0. The sequencing analysis was an exploratory post hoc analysis.

### Sample size and statistical analysis

The objective of this study was to compare the efficacy and safety of CEOP/IVE/GDP alternating regimen with CEOP regimen in newly diagnosed patients with PTCL. For sample size, we estimated that 35% of patients in the CEOP group and 60% of patients in the CEOP/IVE/GDP group would achieve CR. Fifty patients per group were required to show this difference with 5% significance (two-sided) and 73% power, with no plan for interim analysis. The number of patients achieving CR at the end of study was reported by treatment group with 95% CI using normal approximation and compared between groups using logistic regression, with odds ratio (OR [95% CI]) for CEOP/IVE/GDP versus CEOP. Additionally, a logistic regression model was fitted to adjust for the stratification variables, with OR (95% CI) for CEOP/IVE/GDP versus CEOP. For those whose disease could not be evaluated were considered as no responders from statistically conservative consideration.

We planned subgroup analysis to assess treatment response in the predefined subgroups by logistic regression, with results displayed as ORs in a Forest plot. Kaplan-Meier methods were used, and we compared survival between two study groups by a log-rank test. Additionally, a Cox proportional analysis in the intent-to-treatment (ITT) population combining both study groups: age, gender, PTCL subtype, IPI risk group, treatment, Epstein-Barr virus DNA (EBV-DNA), and gene mutations. ITT population was defined in the protocol as all subjects who were randomized to either of the treatment group and accepted at least one dose of treatment. The data analysis was generated using SPSS Statistics version 23 and GraphPad Prism 7.

## Results

### Patient characteristics

**Fig. 1:**
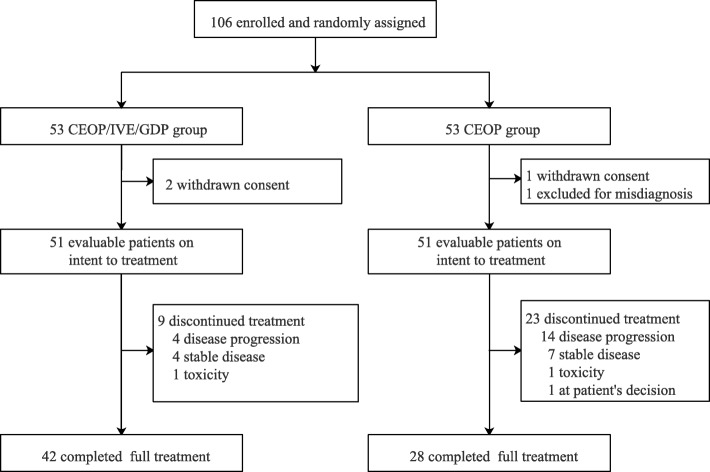
CONSORT diagram of the study. CEOP = cyclophosphamide, epirubicin, vincristine, and prednisone. IVE = ifosmide, epirubicin, and etoposide. GDP = gemcitabine, cisplatin, and dexamethasone

Between September 22, 2015, and December 30, 2018, 106 patients were randomly assigned to two study groups: 53 each to the CEOP/IVE/GDP group and the CEOP group. One patient was excluded because of the change of diagnosis, and 3 withdrew informed consent before study treatment in both groups. Fifty-one patients in each group were included into efficacy and safety analysis as ITT population. Thirty-two patients failed to complete full 6 cycles due to disease progression (18), stable disease (11), toxicity (2), and consent withdrawal (1). In the CEOP/IVE/GDP group, 9 patients discontinued treatment due to failure to response (*n* = 8, including 4 with disease progression and 4 with stable disease at interim evaluation) and toxicity (*n* = 1). Median time from randomization to treatment failure was 3.3 months in patients with disease progression (range from 1.2 to 5.4 months) and 3.0 months in patients with stable disease (range from 2.5 to 3.4 months), respectively. In the CEOP group, 23 discontinued treatment due to failure to response (*n* = 21, including 14 with disease progression and 7 with stable disease at interim evaluation), toxicity (*n* = 1), and consent withdrawal (*n* = 1). Median time from randomization to treatment failure was 3.5 months in patients with disease progression (range from 0.3 to 5.6 months) and 3.2 months in patients with stable disease (range from 2.6 to 3.7 months), respectively. Seventy patients finally completed full 6 cycles of treatment (Fig. 1).

Patient characteristics are summarized in Table 1. No significant difference of baseline characteristics like age, gender, Ann Arbor stage, B symptoms, performance status, serum LDH, EBV-DNA, and IPI risk group was observed between the two study groups. The subtype distribution was as follows: PTCL-NOS (47/102, 46.1%), AITL (34/102, 33.3%), ALCL-ALK negative (16/102, 15.7%), EATL (4/102, 3.9%), HSTL (1/102, 1.0%). The proportion of intermediate- and high-risk patients (i.e., IPI 2–5) was similar and accounted for 70.6% (72/102) of all patients. As of April 25, 2019, median follow-up time was 37.2 months (IQR 34.7–39.7). One hundred and two patients started treatment and six (5.9%) of them went for HSCT, including four autologous HSCT in the CEOP/IVE/GDP group, one autologous HSCT, and one allogenic HSCT in the CEOP group. Among 29 complete responders who did not undergo HSCT, the reasons included patients’ willingness (10/29, 34.5%), age ≥ 65 years (9/29, 31.0%), medical comorbidities (7/29, 24.1%), early relapse (2/29, 6.9%), and stem cell mobilization failure (1/29, 3.4%).

**Table 1:** Baseline characteristics in intent-to-treatment population

	CEOP/IVE/GDP (*N* = 51)	CEOP (*N* = 51)
Age (years; median IQR)	56 (46.0–64.0)	60 (52.0–65.0)
≤ 60 years	33 (65%)	28 (55%)
> 60 years	18 (35%)	23 (45%)
Gender		
Male	34 (67%)	32 (63%)
Female	17 (33%)	19 (37%)
Ann Arbor Stage		
I–II	10 (20%)	9 (18%)
III–IV	41 (80%)	42 (82%)
B symptoms		
Absent	26 (51%)	23 (45%)
Present	25 (49%)	28 (55%)
Performance status		
0–1	46 (90%)	42 (82%)
2	5 (10%)	9 (18%)
Serum LDH		
Normal	25 (49%)	28 (55%)
Elevated	26 (51%)	23 (45%)
EBV-DNA		
Undetectable	35/45 (78%)	30/42 (71%)
Detectable	10/45 (22%)	12/42 (29%)
Extra-nodal involvement		
0–1	34 (67%)	30 (59%)
≥ 2	17 (33%)	21 (41%)
Extra-nodal site		
Skin	4 (8%)	5 (10%)
Gastrointestinal tract	5 (10%)	7 (14%)
Liver	4 (8%)	2 (4%)
Spleen	14 (27%)	14 (27%)
Lung	2 (4%)	3 (6%)
Bone marrow	15 (30%)	21 (41%)
Bone	5 (10%)	8 (16%)
Breast	0	1 (2%)
IPI		
0–1	14 (27%)	16 (31%)
2–3	30 (59%)	26 (51%)
4–5	7 (14%)	9 (18%)

Data are median (IQR), *n* (%), or *n*/*N* (%). *CEOP* cyclophosphamide, epirubicin, vincristine, and prednisone, *IVE* ifosmide, epirubicin, and etoposide, *GDP* gemcitabine, cisplatin, and dexamethasone, *LDH* lactate dehydrogenase, *IPI* International Prognostic Index

### Mutation pattern

**Fig. 2:**
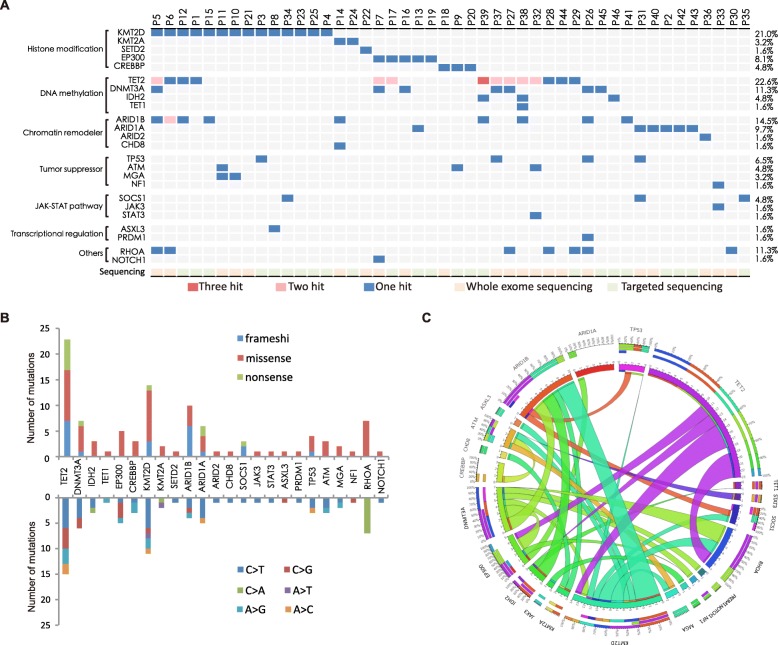
Gene Mutations in peripheral T cell lymphoma. **a** Gene mutations identified by whole exome sequencing and targeted sequencing in 62 patients. The percentage of patients with mutations was listed on the right. The mutations are classified into the categories indicated on the left. **b** Number and type of non-silent somatic mutations presented above, number and percentage of non-silent somatic single-nucleotide variants presented below. **c** Circos diagram presenting correlation between genes

As shown in Fig. 2a, assessed by WES and targeted sequencing, gene mutations were identified in 46 of 62 (71.2%) patients, including histone modification (*KMT2D*, *KMT2A*, *SETD2*, *EP300*, and *CREBBP*), DNA methylation (*TET2*, *DNMT3A*, *IDH2*, and *TET1*), chromatin remodeler (*ARID1B*, *ARID1A*, *ARID2*, and *CHD8*), and tumor suppressor (*TP53*, *ATM*, *MGA*, and *NF1*), JAK-STAT pathway (*SOCS1*, *JAK3*, and *STAT3*), transcriptional regulation (*ASXL3* and *PRDM1*), and other genes (*RHOA* and *NOTCH1*). No mutation of *EZH2*, *KDM6A*, *APC*, *JAK1*, *STAT5B*, *IL2RG DDX3X*, *BCOR*, or *FYN* was detected. Most of the somatic mutations were missense mutations (*n* = 70), followed by frameshift mutations (*n* = 21) and nonsense mutations (*n* = 11) (Fig. 2b). We observed a preference for C>T alterations analogous to the somatic single-nucleotide variation spectrum in other cancers (Fig. 2b). Histone modification gene mutations were mutually exclusive of each other, suggesting that they might be involved in distinct biological processes (Fig. 2c).

In terms of gene category, histone modification genes were most frequently mutated, namely *KMT2D* (14/62, 22.6%), *KMT2A* (2/62, 3.2%), *SETD2* (1/62, 1.6%), *EP300* (5/62, 8.1%), and *CREBBP* (3/62, 4.8%). DNA methylation gene mutations occurred in *TET2* (14/62, 22.6%), *DNMT3A* (7/62, 11.3%), *IDH2* (3/62, 4.8%), and *TET1* (1/62, 1.6%). Chromatin remodeler gene mutations occurred in *ARID1B* (9/62, 14.5%), *ARID1A* (6/62, 9.7%), *ARID2* (1/62, 1.8%), and *CHD8* (1/62, 1.8%). Tumor suppressor gene mutations occurred in *TP53* (4/62, 6.5%), *ATM* (3/62, 4.8%), *MGA* (2/62, 3.2%), and *NF1* (1/62, 1.6%). JAK-STAT pathway gene mutations occurred in *SOCS1* (3/62, 4.8%), *JAK3* (1/62, 1.6%), and *STAT3* (1/62, 1.6%). Transcriptional regulation gene mutations occurred in *ASXL3* (1/62, 1.6%) and *PRDM1* (1/62. 1.6%). Other mutations occurred in *RHOA* (7/62, 11.3%) and *NOTCH1* (1/62, 1.6%), respectively.

According to histological subtypes, the most frequent mutated gene was *KMT2D* (7/34, 20.6%) in PTCL-NOS, *TET2* (9/18, 50.0%) in AITL, and *DNMT3A* (2/7, 28.6%) in ALCL-ALK negative. *EP300*, *TET2*, and *ARID1B* mutations had significantly higher proportion in AITL than in PTCL-NOS (22.2% vs. 2.9%, *p* = 0.043, 50.0% vs. 14.7%, *p* = 0.010, 33.3% vs. 2.9%, *p* = 0.003, respectively).

### Response to treatment and prognosis

Treatment response rate was summarized in Table 2. The CRR at EOT in the CEOP/IVE/GDP group was similar to that in the CEOP group (37.3% vs. 31.4%, OR 0.84, 95% CI 0.49–1.44; *p* = 0.532), whereas ORR in the CEOP/IVE/GDP group was superior to the CEOP group (72.5% vs. 49.0%, OR 0.68, 95% CI 0.48–0.93; *p* = 0.015). CR status at EOT was strongly associated with long-term survival time (median PFS not reached in the CR group vs. 13.8 months [95% CI 10.34–17.07] in the PR group; HR 0.25, 95% CI 0.12–0.52; *p* < 0.001; median OS not reached in CR group vs. 24.3 months [17.9–30.7] in PR group; HR 0.23, 95% CI 0.10–0.55; *p* < 0.001; Additional file 1: Figure S1).

**Table 2:** Treatment response

	CEOP/IVE/GDP (*N* = 51) ^*^	CEOP (*N* = 51) ^*^	*p* value
Response at interim			
Complete response	17 (33.3%)	18 (35.3%)	0.834
Partial response	26 (51.0%)	13 (25.5%)	0.008
Stable disease	4 (7.8%)	7 (13.7%)	
Disease progression	3 (5.9%)	11 (21.6%)	
Overall response	43 (84.3%)	31 (60.8%)	0.008
Response at EOT			
Complete response	19 (37.3%)	16 (31.4%)	0.532
Partial response	18 (35.3%)	9 (17.6%)	0.042
Stable disease	4 (7.8%)	7 (13.7%)	
Disease progression	9 (17.6%)	17 (33.3%)	
Overall response	37 (72.5%)	25 (49. 0%)	0.015

*EOT* end of treatment

*One patient in the CEOP/IVE/GDP group could not be evaluated due to toxicity (*n* = 1). Two patients in the CEOP group could not be evaluated due to toxicity (*n* = 1) and consent withdrawal (*n* = 1). All the three patients were considered as no responders from statistically conservative consideration

As for interim evaluation, CRR in the CEOP/IVE/GDP group was similar to that in the CEOP group (33.3% vs. 35.3%, OR 1.06, 95% CI 0.62–1.81; *p* = 0.834), whereas ORR in the CEOP/IVE/GDP group was superior to the CEOP group (84.3% vs. 60.8%, OR 0.72, 95% CI 0.55–0.92; *p* = 0.008). CR status at interim evaluation was also associated with long-term survival time (median PFS not reached in the CR group vs. 10.7 months [95% CI 6.34–14.99] in the PR group; HR 0.29, 95% CI 0.16–0.55; *p* < 0.001; median OS not reached in CR group vs. 23.8 months [18.4–29.2] in PR group; HR 0.29, 95% CI 0.15–0.56; *p* < 0.001; Additional file 1: Figure S1). Seventy-four (72.5%) patients achieved response at interim evaluation in the whole study population. Among 35 CR patients at interim evaluation, 32 (91.4%) patients remained CR and 3 (8.6%) had progressive disease at EOT. Of the 39 PR patients, 3 (7.7%) patients achieved CR, 27 (69.2%) patients remained PR, and 9 (23.1%) had progressive disease at EOT.

**Fig. 3:**
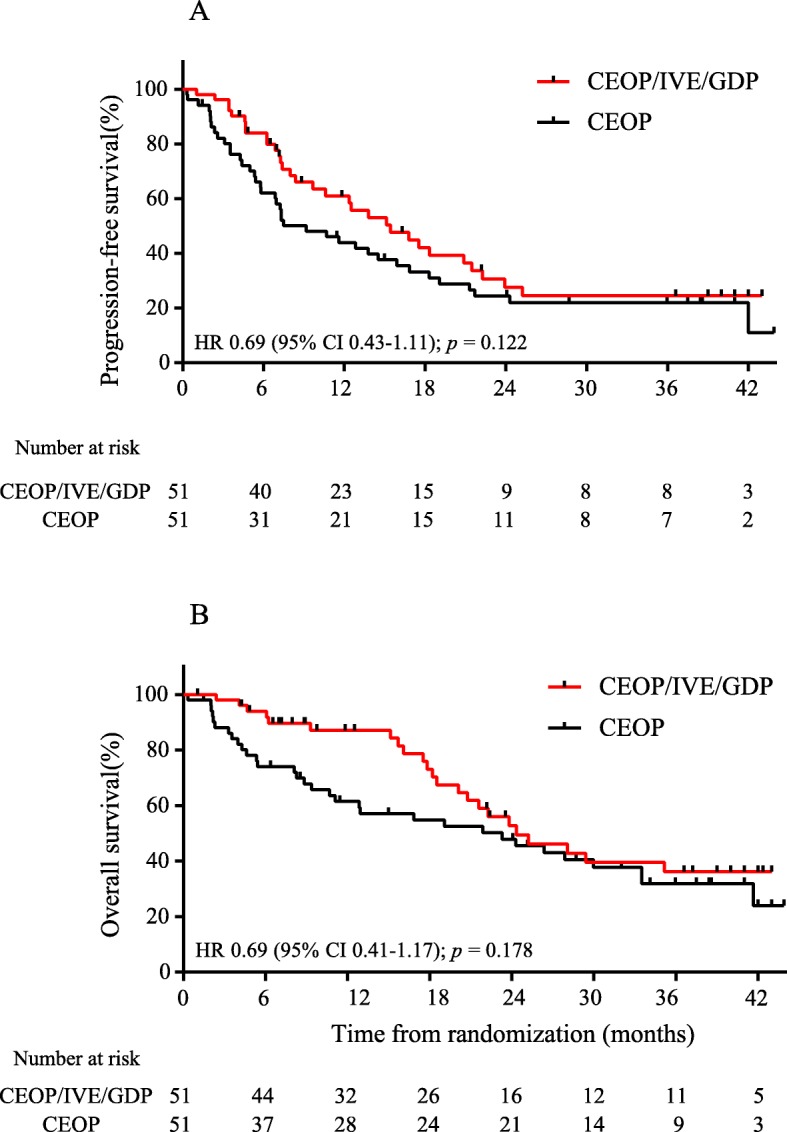
Treatment response and survival outcomes. Kaplan-Meier curves showed (**a**) progression-free survival and (**b**) overall survival of the CEOP/IVE/GDP group and of the CEOP group. HR = hazard ratio

The median PFS of the CEOP/IVE/GDP group was similar to that of the CEOP group (15.4 months [95% CI 9.8–21.1] vs. 9.2 months [4.2–14.2], HR 0.69, 95% CI 0.43–1.11, *p* = 0.122, Fig. 3a), corresponding to a 2-year PFS rate of 25.0% (95% CI 11.2–38.8) for the CEOP/IVE/GDP group and 25.4% (12.7–38.1) for the CEOP group. The median OS was also similar in both groups (24.3 months [95% CI 17.0–31.6] vs 21.9 months [7.3–36.2], HR 0.69, 95% CI 0.41–1.17, *p* = 0.178, Fig. 3b), corresponding to a 2-year OS rate of 49.2% (95% CI 32.7–65.7) for the CEOP/IVE/GDP group and 47.1% (32.8–61.4) for the CEOP group.

### Factors impacting response to treatment

**Fig. 4:**
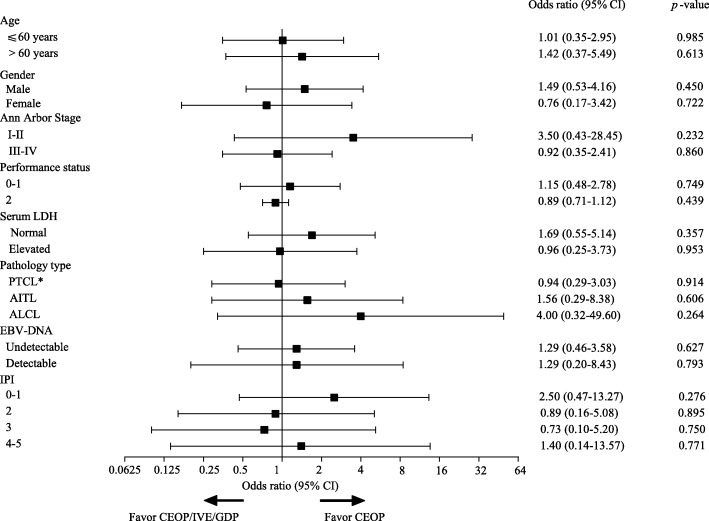
Subgroup analysis for complete response at end of treatment. LDH = lactate dehydrogenase, IPI = International Prognostic Index. *PTCL subtype includes PTCL-NOS and other types

Post hoc subgroup analysis of CRR at EOT showed no difference in CRR between two study groups regarding age, gender, sex, Ann Arbor stage, performance status, serum LDH, PTCL subtype, EBV-DNA, and IPI risk group (Fig. 4). We also investigated the predictive ability of IPI, NCCN-IPI, and PIT model and found similar c-index for PFS (IPI 0.61, NCCN-IPI 0.57, PIT 0.60) and OS (IPI 0.68, NCCN-IPI 0.63, PIT 0.66). Interestingly, 11 patients of low-risk NCCN-IPI showed excellent prognosis with 2-year OS > 80% without HSCT (only 1 out of 11 patients died at 16.1 months due to disease progression, Additional file 1: Figure S3).

**Fig. 5:**
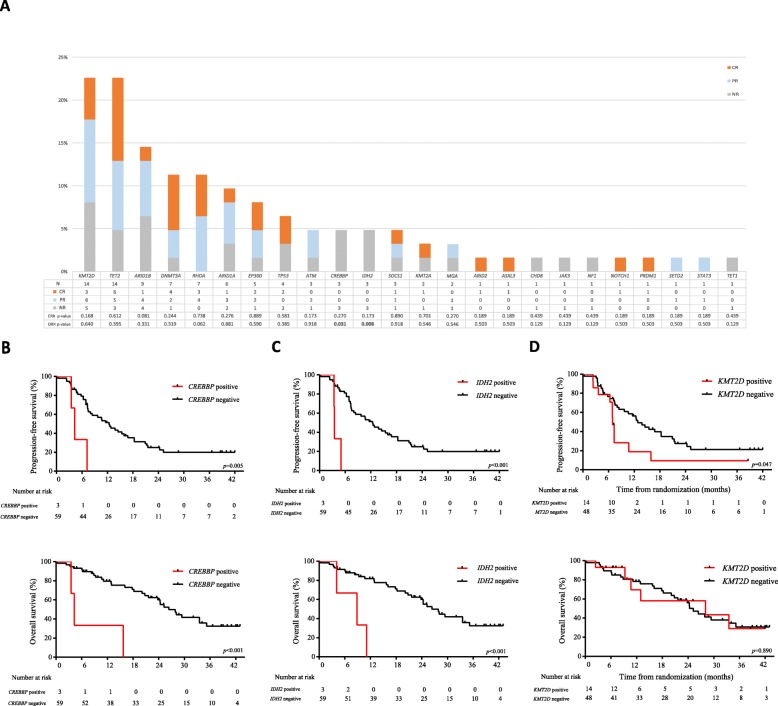
Gene mutations and treatment response/survival outcomes. **a** Relation between treatment response (including complete response, partial response, and no response) and gene mutation profile. **b**–**d** Kaplan-Meier curves showed progression-free survival (upper panel) and overall survival (lower panel) of PTCL patients, according to the mutation status of *CREBBP* (**b**), *IDH2* (**c**), and *KMT2D* (**d**)

The relationship of the recurrent mutated genes with the response status at EOT was shown in Fig. 5a. Among all, *CREBBP* and *IDH2* gene mutations were significantly associated with fewer response to treatment at EOT, as compared to those without mutation (ORR 0% vs. 72.9%, *p* = 0.031 and 0% vs. 72.9%, *p* = 0.008, respectively). Moreover, *KMT2D* mutations were observed in 14 patients, of which only 3 patients with CR at EOT, and 6 patients with PR and 5 with NR. Comparing with those without mutation, patients with *KMT2D* mutation had higher proportion of no response and early progression after PR at interim evaluation (57.1% vs. 25.0%, *p* = 0.048). Among 7 patients who had PR at interim evaluation but PD at EOT, 3 had *KMT2D* mutation, 2 had *ARID1B* mutation, and 1 had *CREBBP* mutation.

### Factors impacting prognosis

Clinicopathological parameters and recurrent mutated genes were assessed by univariable analysis. Non-ALCL subtype, intermediate/high-risk IPI, and detectable EBV-DNA, as well as mutations in *CREBBP*, *IDH2*, and *ARID1B*, were associated with inferior PFS and OS, while mutations in *KMT2D* were associated only with inferior PFS (Additional file 1: Table S3). By multivariate analysis, mutations in *CREBBP*, *IDH2*, and *KMT2D* were independent factors predicting poor PFS, while *CREBBP* and *IDH2* were independent factors predicting poor OS (Additional file 1: Table S4). Median PFS and OS in patients with *CREBBP* mutation was significantly shorter than those without *CREBBP* mutation (PFS, 4.3 months [3.0–5.6] vs. 12.4 months [95% CI 8.7–16.0], HR 4.89, 95% CI 1.41–16.89, *p* = 0.005, OS, 4.3 months [3.1–5.5] vs. 26.4 months [95% CI 21.1–31.6], HR 7.27, 95% CI 2.04–25.84, *p* < 0.001, Fig. 5b). Similar results of median PFS and OS were also observed between patients with or without *IDH2* mutation (PFS, 3.5 months [3.3–3.7] vs. 12.4 months [95% CI 8.7–16.0], HR 8.21, 95% CI 2.21–30.42, *p* < 0.001, OS, 8.9 months [1.2–16.4] vs. 26.4 months [95% CI 21.1–31.6], HR 7.29, 95% CI 1.99–26.74, *p* < 0.001, Fig. 5c). According to *KMT2D* mutation, the median PFS in patients with *KMT2D* mutation was significantly reduced, as compared to those without *KMT2D* mutation (6.9 months [6.5–7.3] vs. 12.8 months [95% CI 9.2–16.4], HR 2.00, 95% CI 0.99–4.01, *p* = 0.047, Fig. 5d). Among eight patients with *CREBBP* or *EP300* mutation, 2 of them received and responded to histone deaceylase (HDAC) inhibitor chidamide combined with chemotherapy as the second-line treatment (1 achieved PR after 4 months of treatment and remained alive, the other achieved PR after 2 months and died of disease progression after 4 months). Among 14 patients with *KMT2D* mutation, 8 of them received chidamide as the second-line treatment, either alone (*n* = 3) or in combination with chemotherapy (*n* = 5). Two patients achieved CR, three patients achieved PR, one patient had stable disease, and two patients had disease progression.

In terms of IPI, NCCN-IPI, and PIT, CEOP/IVE/GDP could improve survival outcomes in high-risk patients. Briefly, in patients with IPI of 4–5, increased median OS was observed in the CEOP/IVE/GDP group (20.1 months [95% CI 15.4–24.8] vs. 4.6 months [0.0–11.3], HR 0.28, 95% CI 0.09–0.90, *p* = 0.012). Similar results of OS were observed in NCCN-IPI of 4–6 (23.8 months [95% CI 18.6–29.0] vs. 9.4 months [5.8–12.9], HR 0.44, 95% CI 0.22–0.89, *p* = 0.028) and PIT of 3–4 (20.1 months [95% CI 15.3–24.9] vs. 4.6 months [0.0–13.8], HR 0.31, 95% CI 0.11–0.93, *p* = 0.021). Meanwhile, increased median PFS was also observed in NCCN-IPI of 4–6 (16.8 months [95% CI 10.1–23.5] vs. 5.8 months [3.1–8.5], HR 0.46, 95% CI 0.24–0.89, *p* = 0.024) and PIT of 3–4 (15.4 months [95% CI 7.7–23.2] vs. 2.6 months [0.3–4.9], HR 0.32, 95% CI 0.11–0.96, *p* = 0.026, Additional file 1: Table S5). In low-risk patients under all prognostic indexes, more patients could achieve CR at EOT (CRR 60.0% in patients with IPI of 0–1, 63.6% in NCCN-IPI of 0–1, 54.2% in PIT of 0, Additional file 1: Figure S2).

### Toxicities

Adverse events of both hematological and non-hematological toxicity were listed in Table 3. Safety was assessed in 102 patients who received at least one dose of study treatment. No statistical significance on hematological adverse events was observed between the two study groups. Neutropenia was the most common event in both groups (82% in the CEOP/IVE/GDP group and 86% in the CEOP group). One patient in the CEOP group died from severe infection after 1 cycle and one patient in the CEOP/IVE/GDP group was unable to continue treatment due to pulmonary infection after 2 cycles. For non-hematological adverse events, CEOP/IVE/GDP was associated with more headaches of all grades (27% in the CEOP/IVE/GDP group and 12% in the CEOP group).

**Table 3:** Incidence of adverse events

	CEOP/IVE/GDP (*N* = 51)			CEOP (*N* = 51)		
	Grade 1–2	Grade 3	Grade 4	Grade 1–2	Grade 3	Grade 4
Hematological event						
Neutropenia	11 (22%)	9 (18%)	22 (43%)	12 (24%)	9 (18%)	23 (45%)
Thrombocytopenia	12 (24%)	2 (4%)	2 (4%)	10 (20%)	1 (2%)	3 (6%)
Anemia	34 (67%)	4 (8%)	1 (2%)	31 (61%)	5 (10%)	0
Febrile neutropenia	/	10 (20%)	0	/	12 (24%)	2 (4%)
Non-hematological events						
Infection*	9 (18%)	4 (8%)	2 (4%)	10 (20%)	6 (12%)	1 (2%)
Nausea or vomiting	15 (29%)	2 (4%)	0	19 (37%)	1 (2%)	0
ALT or AST increase	9 (18%)	2 (4%)	0	8 (16%)	1 (2%)	0
Mucositis	13 (25%)	0	0	15 (29%)	1 (2%)	0
Fatigue	37 (73%)	1 (2%)	0	34 (67%)	1 (2%)	0
Headache	13 (25%)	1 (2%)	0	5 (10%)	1 (2%)	0

Data are *n* (%). All patients who received at least one dose of study drug were included in the safety analysis. *One patient in CEOP group died from severe infection after 1 cycle of treatment. *ALT* alanine aminotransferase, *AST* aspartate transaminase

## Discussion

To our knowledge, this is the first randomized clinical trial to evaluate the efficacy and safety of alternating regimen in treating newly diagnosed patients with PTCL. However, CEOP/IVE/GDP alternating regimen failed to meet the primary endpoint and showed no remission or survival advantage when comparing with CEOP. ALCL-ALK positive that was generally associated with good response to CHOP-based regimen had been excluded according to the study protocol, which could explain the short OS tendency of this study.

Multi-drug combinations with different mechanisms of action may alter the clinical outcomes in lymphoma. Gemcitabine is not a substrate for MDR-mediated efflux and proves effective in PTCL [4, 19–22]. A retrospective study comparing CHOP, CHOP-E, and GDP as first-line treatment of PTCL in 102 patients showed that GDP improves CRR, ORR, and OS [4]. Another randomized controlled trial also found that GDP with thalidomide is superior to standard CHOP in 103 newly diagnosed patients with PTCL for response rate and survival time [21]. However, a recent randomized trial in 87 patients with PTCL indicated that GEM-P (gemcitabine, cisplatin, methylprednisolone) is similar to CHOP in the first-line treatment [23]. As for IVE, it shows promising anti-tumor activity in patients with refractory/relapsed lymphomas [5–10] and is introduced as an effective front-line treatment in combination with CHOP and MTX in EATL [11]. Based on the above clinical data, we combined these regimens and conducted a phase 2, multicenter, randomized, controlled trial, aiming to increase efficacy in PTCL. Nevertheless, remission and prognostic status of PTCL may not be altered by chemotherapy in an alternating manner. Meanwhile, it is worth pointing out that response at EOT was a strong prognostic factor of both PFS and OS. Consistent with a previous study showing that complete response is a predictor of favorable outcome in PTCL [24], intensive strategies such as HSCT consolidation once CR was achieved are helpful to sustain long-term response and survival.

In terms of prognostic index, IPI, NCCN-IPI, and PIT had comparable capability for risk stratification. For low-risk patients, CRR was much higher under all three prognostic indexes. Of note, NCCN-IPI can identify very low-risk population, with 2-year OS > 80% without HSCT, in accordance with our previous study [25]. Interestingly, for high-risk patients, CEOP/IVE/GDP alternating regimen was found to prolong PFS and OS, as risk stratified by NCCN-IPI (4–6) and PIT (3–4). Further randomized clinical trials are needed to carry out in these high-risk subgroups of PTCL using alternating chemotherapy followed by HSCT.

Importantly, using WES and targeted sequencing, we identified mutations in epigenetic regulators that were closely related to disease progression of PTCL. It is reported that histone methylation gene *KMT2D* mutations correlate with shorter OS in Epstein-Barr virus-associated natural killer-cell lymphoma [26] and are the most frequent relapse-specific events in diffuse large B cell lymphoma (DLBCL) [27]. Presence of histone acetylation gene *CREBBP* mutations is an independent prognostic factor in DLBCL by mutational analysis of the SAKK 38/07 prospective cohort [28]. *IDH2* mutations induce histone and DNA hypermethylation and define a unique subgroup of PTCL with follicular T-helper-like phenotype [29]. *ARID1B*, a subunit of the SWI/SNF chromatin complex, is also frequently mutated in aggressive lymphomas, including hepatosplenic T cell lymphoma [30] and DLBCL [31]. Therefore, we further confirmed that epigenetic factors can function as predictive biomarkers of inferior prognosis in PTCL.

Epigenetic regulator gene mutations render PTCL resistant to chemotherapy, but sensitive to targeted agents. Experimentally, HDAC inhibitor chidamide significantly inhibited the growth of *EP300*-mutated T-lymphoma cells and *KMT2D*-mutated T-lymphoma cells when combined with the hypomethylating agent decitabine [12]. Clinically, a phase 1 study of HDAC inhibitor romidepsin and hypomethylating agent 5-azacytidine exhibited marked activity in patients with PTCL, with ORR and CR of 73% and 55%, respectively [32]. Therapeutic efficacy of novel small molecules like *IDH2* inhibitors targeting *IDH2*, or modulators of the *SWI/SNF* chromatin complex targeting *ARID1B* could also be explored in PTCL. In our study, patients bearing *CREBBP*, *EP300*, or *KMT2D* may respond to epigenetic agent chidamide, either alone or in combination with chemotherapy. Although these results should be further investigated in a larger cohort of patients, detection of epigenetic gene mutations may be helpful for selection of patients to targeted therapeutic agents and eventually improve the clinical outcome of PTCL in the era of precision medicine.

Some limitations in our study should be acknowledged. Firstly, although the majority of the patients were evaluated by PET-CT, methods of CT response assessment were also included. Secondly, maintenance therapy was not pre-specified and the proportion of patients undergoing HSCT was limited.

## Conclusions

Alternative chemotherapy regimen failed to demonstrate an advantage in CRR and survival time, as compared to standard chemotherapy in PTCL. Future clinical trials should aim to develop alternative regimens targeting the biology of the disease as demonstrated by recurrent mutations in epigenetic factors and histone modification genes.

## Supplementary information

Additional file 1. Supplementary figures and tables.

Additional file 2. CONSORT Checklist and CONSORT Extension for Abstract Checklist.

## Data Availability

The datasets used and/or analyzed during the current study are available in Mendeley Data through the DOI 10.17632/jcp3kc4tzx.1 [16] and NODE (http://www.biosino.org/node) by searching OEP000795 or through the URL: http://www.biosino.org/node/project/detail/OEP000795 [17].

## Abbreviations

AITL: Angioimmunoblastic T cell lymphoma
ALCL: Anaplastic large cell lymphoma
ALK: Anaplastic lymphoma kinase
CBC: Complete blood cell count
CEOP: Cyclophosphamide, epirubicin, vincristine, prednisone
CHOP: Cyclophosphamide, doxorubicin, vincristine, prednisone
CRR: Complete response rate
CTCAE: Common Terminology Criteria for Adverse Events
DLBCL: Diffuse large B cell lymphoma
EATL: Enteropathy-associated T cell lymphoma
EBV-DNA: Epstein-Barr virus DNA
EOT: End of treatment
GDP: Gemcitabine, cisplatin, dexamethasone
HBV-DNA: Hepatitis B virus DNA
HDAC: Histone deaceylase
HSCT: Hematopoietic stem cell transplantation
HSTL: Hepatosplenic T cell lymphoma
IPI: International Prognostic Index
IVE: Ifosmide, epirubicin, etoposide
IQR: Interquartile range
LDH: Lactate dehydrogenase
NCCN: National Comprehensive Cancer Network
NHL: Non-Hodgkin lymphoma
ORR: Overall response rate
OS: Overall survival
PD: Progressive disease
PET-CT: Positron emission tomography-computed tomography
PFS: Progression-free survival
PIT: Prognostic Index for T cell lymphoma
PR: Partial response
PTCL-NOS: Peripheral T cell lymphoma-not otherwise specified
WES: Whole exome sequencing
WHO: World Health Organization
