# Supplementary material for: CEOP/IVE/GDP alternating regimen compared with CEOP as the first-line therapy for newly diagnosed patients with peripheral T cell lymphoma: results from a phase 2, multicenter, randomized, controlled clinical trial
Source: Genome Med. 2020 Apr 30;12:41. doi: 10.1186/s13073-020-00739-0 (PMC7191773; doi:10.1186/s13073-020-00739-0)
Supplement: Supplementary file 1 — Supplementary figures and tables. [file 13073_2020_739_MOESM1_ESM.pdf]

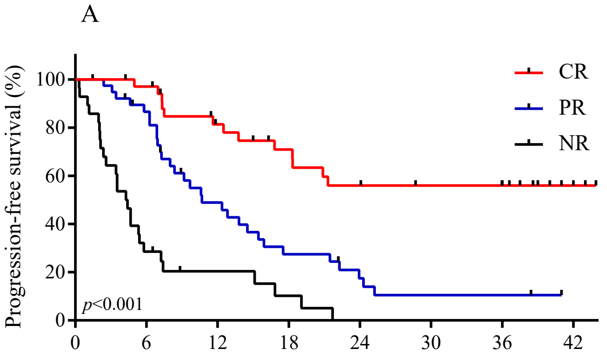

Number at risk

CR	35	33	24	19	14	13	12	4
PR	39	31	16	9	5	3	3	2
NR	28	8	4	2	0	0	0	0

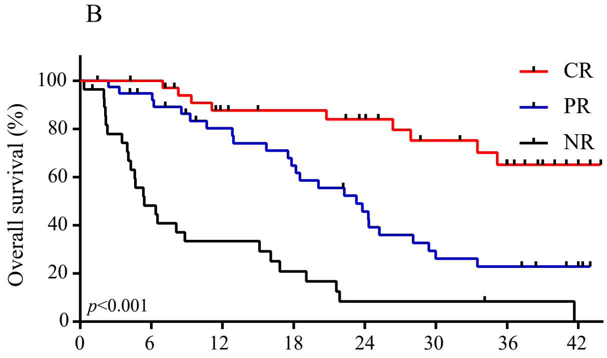

Number at risk

CR	35	34	26	24	21	16	12	5
PR	39	34	26	21	14	8	7	3
NR	28	13	8	5	2	2	1	0

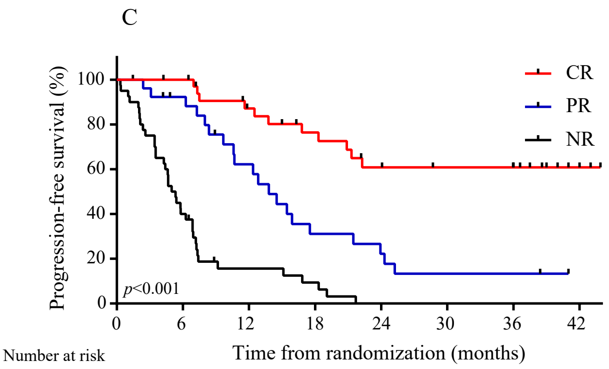

Number at risk

CR	35	34	25	20	15	13	12	5
PR	27	22	14	7	5	3	3	2
NR	40	16	5	1	0	0	0	0

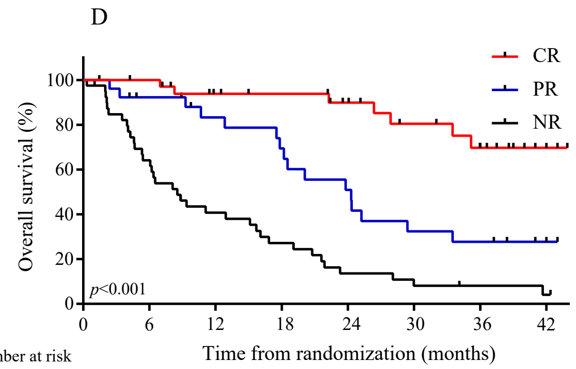

Number at risk

CR	35	34	27	25	21	12	11	5
PR	27	22	18	15	11	6	6	2
NR	40	25	15	10	5	2	5	1

Figure S1. Response and survival outcomes at interim and final evaluation

Kaplan Meier curves showed (A) progression-free survival and (B) overall survival in terms of response at interim evaluation, (C) progression-free survival and (D) overall survival in terms of response at EOT. CR = complete response, PR = partial response, NR = no response, EOT = end of treatment.

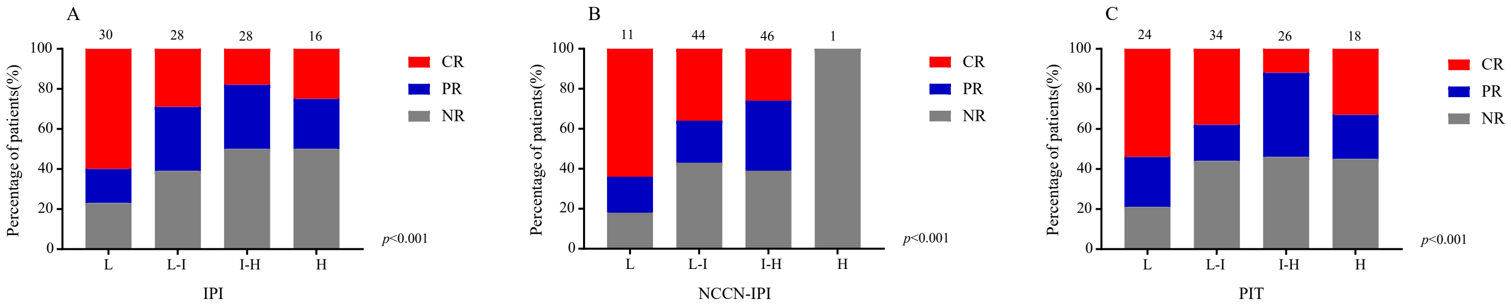

Figure S2. Response at EOT and prognostic index

Response according to (A) IPI, (B) NCCN-IPI, (C) PIT prognostic system. CR=complete response, PR=partial response, NR=no response, L=Low risk, L-I=Low-intermediate risk, I-H=Intermediate-high risk, H=High risk, IPI=International prognostic index, NCCN-IPI=National Comprehensive Cancer Network- International prognostic index, PIT = Prognostic Index for T cell lymphoma.

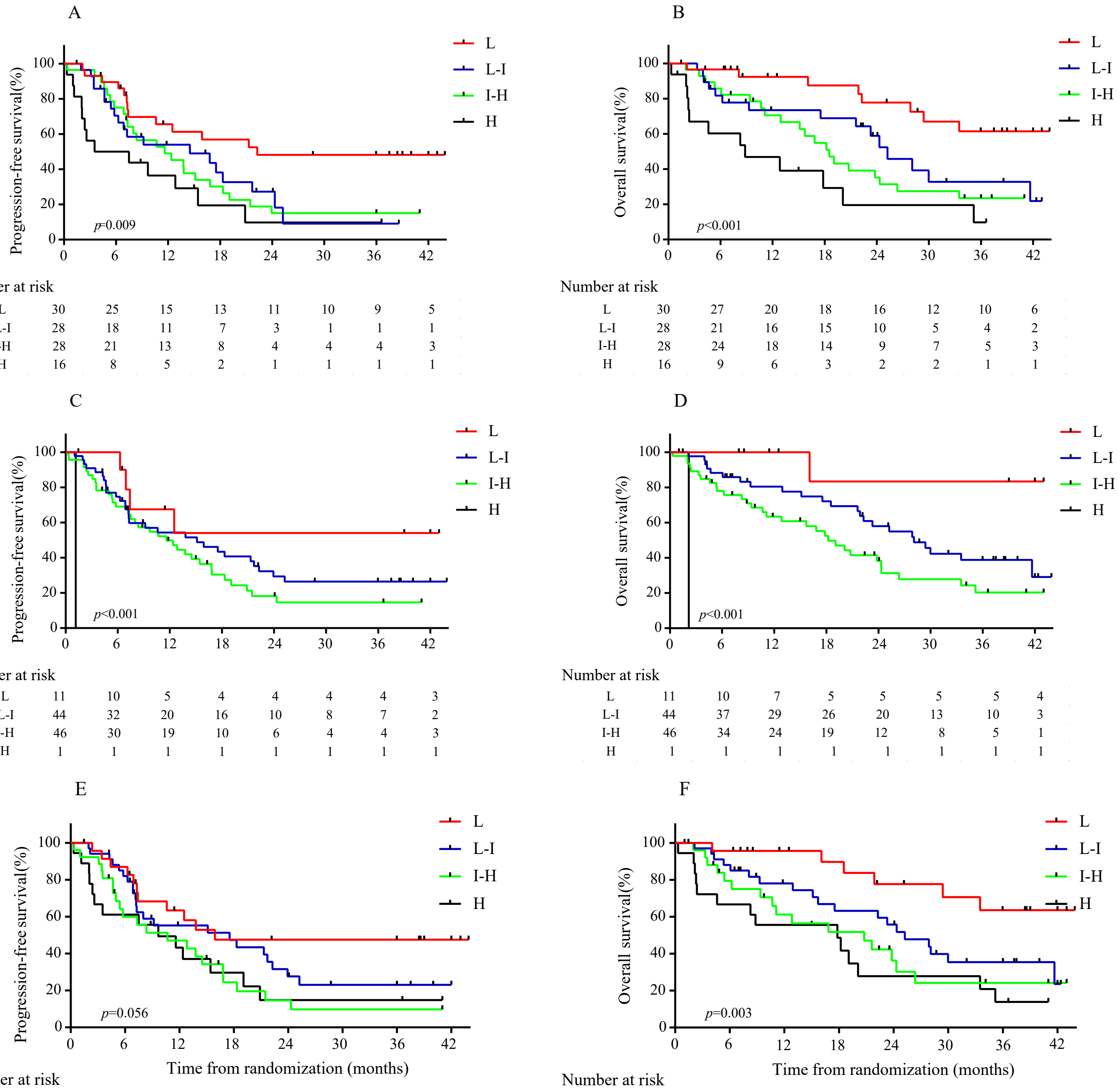

Number at risk (A)

L	30	25	15	13	11	10	9	5
L-I	28	18	11	7	3	1	1	1
I-H	28	21	13	8	4	4	4	3
H	16	8	5	2	1	1	1	1

Number at risk (B)

L	30	27	20	18	16	12	10	6
L-I	28	21	16	15	10	5	4	2
I-H	28	24	18	14	9	7	5	3
H	16	9	6	3	2	2	1	1

Number at risk (C)

L	11	10	5	4	4	4	4	3
L-I	44	32	20	16	10	8	7	2
I-H	46	30	19	10	6	4	4	3
H	1	1	1	1	1	1	1	1

Number at risk (D)

L	11	10	7	5	5	5	5	4
L-I	44	37	29	26	20	13	10	3
I-H	46	34	24	19	12	8	5	1
H	1	1	1	1	1	1	1	1

Number at risk (E)

L	24	20	12	9	8	7	7	4
L-I	34	27	14	12	6	4	3	1
I-H	26	14	11	5	3	2	2	2
H	18	11	7	4	2	2	2	1

Number at risk (F)

L	24	22	17	15	12	10	8	5
L-I	34	29	21	17	15	8	7	2
I-H	26	18	13	11	6	4	3	1
H	18	12	9	6	4	4	2	1

Figure S3. Survival curves and prognostic index
Survival curves according to (A) IPI, (B) NCCN-IPI and (C) PIT prognostic systems

Table S1. Recruiting centers

Site	P.I.	Patients recruited (*N*)
State Key Laboratory of Medical Genomics, Shanghai Institute of Hematology, Shanghai Rui Jin Hospital	Weili Zhao	46
Department of Hematology, Shandong Provincial Hospital Affiliated to Shandong University, Jinan, China	Xin Wang	24
Fujian Institute of Hematology, Fujian Medical University Union Hospital, Fuzhou, China	Jianda Hu	23
Department of Hematology, The Affiliated Cancer Hospital of Zhengzhou University, Henan Cancer Hospital, Zhengzhou, China	Yongping Song	13

P. I. =Principle Investigator

Table S2. Mutation information of 62 patients with PTCL

Category	Genes	Pt No.	Sequencing	Transcription ID	Ref	Alt	AAChange.refGene	ExonicFunc.refGene
Histone modification	*KMT2D*	P1	Target	NM_003482	G	A	p.R3508Q	nonsynonymous
	KMT2D	P3	Target	NM_003482	G	-	p.P2738fs	frameshift
	KMT2D	P4	Target	NM_003482	A	G	p.T2228A	nonsynonymous
	KMT2D	P5	WES	NM_003482	C	T	p.R5224H	nonsynonymous
	KMT2D	P6	WES	NM_003482	C	T	p.R5224H	nonsynonymous
	KMT2D	P8	Target	NM_003482	C	T	p.P2382S	nonsynonymous
	KMT2D	P10	WES	NM_003482	T	C	p.T2228A	nonsynonymous
	KMT2D	P11	WES	NM_003482	A	C	p.V1561G	nonsynonymous
	KMT2D	P12	Target	NM_003482	T	A	p.L4551X	stopgain
	KMT2D	P15	Target	NM_003482	A	AGCT	p.Q3919fs	frameshift
	KMT2D	P21	WES	NM_003482	C	G	p.A3676P	nonsynonymous
	KMT2D	P23	Target	NM_003482	C	T	p.S186L	nonsynonymous
	KMT2D	P25	Target	NM_003482	G	A	p.G2881S	nonsynonymous
	KMT2D	P34	Target	NM_003482	T	TTGC	p.0	frameshift
	KMT2A	P14	WES	NM_001197104	T	A	p.N2347K	nonsynonymous
	KMT2A	P24	Target	NM_001197104	C	A	p.P3914H	nonsynonymous
	SETD2	P22	WES	NM_001349370	C	T	p.S2278N	nonsynonymous
	EP300	P7	WES	NM_001429	G	C	p.Q63H	nonsynonymous
	EP300	P13	Target	NM_001429	A	G	p.I997V	nonsynonymous
	EP300	P16	WES	NM_001429	C	T	p.P847S	nonsynonymous
	EP300	P17	Target	NM_001429	G	C	p.Q1455H	nonsynonymous
	EP300	P19	Target	NM_001429	G	C	p.W1466C	nonsynonymous
	CREBBP	P9	Target	NM_004380	A	G	p.Y1450C	nonsynonymous
	CREBBP	P18	WES	NM_001079846	A	G	p.M250T	nonsynonymous
	CREBBP	P20	Target	NM_004380	C	T	p.R1446C.	nonsynonymous
DNA	*TET2*	P1	Target	NM_001127208	T	C	p.F125S	nonsynonymous

methylation	*TET2*	P5	WES	NM_001127208	C	T	p.Q960X	stopgain
	TET2	P5	WES	NM_001127208	-	A	p.N837fs	frameshift
	TET2	P6	WES	NM_001127208	T	G	p.Y1659X	stopgain
	TET2	P7	WES	NM_001127208	C	T	p.H1380Y	nonsynonymous
	TET2	P7	WES	NM_001127208	A	C	p.N1387H	nonsynonymous
	TET2	P12	Target	NM_001127208	C	G	p.P29R	nonsynonymous
	TET2	P17	Target	NM_001127208	C	G	p.R1216G	nonsynonymous
	TET2	P17	Target	NM_001127208	G	C	p.C1289S	nonsynonymous
	TET2	P27	WES	NM_001127208	C	-	p.Y1421X	stopgain
	TET2	P27	WES	NM_001127208	AA	-	p.Q770fs	frameshift
	TET2	P28	WES	NM_001127208	CAGT	-	p.P1520fs	frameshift
	TET2	P29	Target	NM_001127208	4634delA	4634delA	p.Q1545fs	frameshift
	TET2	P32	WES	NM_001127208	A	G	p.K1299E	nonsynonymous
	TET2	P32	WES	NM_001127208	-	G	p.D1143fs	frameshift
	TET2	P37	WES	NM_001127208	C	G	p.Y1679X	stopgain
	TET2	P37	WES	NM_001127208	-	A	p.I750fs	frameshift
	TET2	P38	WES	NM_001127208	C	T	p.A1355V	nonsynonymous
	TET2	P38	WES	NM_001127208	A	G	p.K1310E	nonsynonymous
	TET2	P39	Target	NM_001127208	C	T	p.Q790X	stopgain
	TET2	P39	Target	NM_001127208	C	T	p.Q916X	stopgain
	TET2	P39	Target	NM_001127208	C	T	p.T444I	nonsynonymous
	TET2	P44	WES	NM_001127208	A	-	p.S1059fs	frameshift
	DNMT3A	P5	WES	NM_001320893	ATCATCTC CCTCCTTG GGCCGCG CATC	-	p.588_596del	frameshift
	DNMT3A	P7	WES	NM_001320893	G	C	p.R619G	nonsynonymous
	DNMT3A	P16	WES	NM_001320892	C	T	p.G10R	nonsynonymous
	DNMT3A	P26	WES	NM_001320893	C	T	p.R730H	nonsynonymous

	DNMT3A	P27	WES	NM_001320893	C	G	p.R479T	nonsynonymous
	DNMT3A	P37	WES	NM_001320893	C	T	p.W449X	stopgain
	DNMT3A	P45	Target	NM_022552	C	T	p.R882C	nonsynonymous
	IDH2	P38	WES	NM_001290114	C	T	p.R42K	nonsynonymous
	IDH2	P39	Target	NM_002168	G	T	p.R172M	nonsynonymous
	IDH2	P46	Target	NM_002168	G	A	p.R172K	nonsynonymous
	TET1	P38	WES	NM_030625	T	C	p.I173T	nonsynonymous
Chromatin remodeler	*ARID1B*	P5	WES	NM_001346813	GGAGGAG GAGGA	-	p.321_324del	frameshift
	ARID1B	P6	WES	NM_001346813	-	GC	p.Q121fs	frameshift
	ARID1B	P6	WES	NM_001346813	-	ACAG	p.Q121fs	frameshift
	ARID1B	P12	Target	NM_017519	G	A	p.V1503I	nonsynonymous
	ARID1B	P14	WES	NM_001346813	-	GGC	p.V339delinsVA	frameshift
	ARID1B	P15	Target	NM_001346813	CAG	-	p.114_114del	frameshift
	ARID1B	P26	WES	NM_017519	C	G	p.Q1393E	nonsynonymous
	ARID1B	P38	WES	NM_001346813	-	AGGAGC	p.G327delinsG GA	frameshift
	ARID1B	P39	Target	NM_020732	A	G	p.M1578V	nonsynonymous
	ARID1B	P41	Target	NM_020732	C	T	p.P737S	nonsynonymous
	ARID1A	P2	Target	NM_006015	G	A	p.A339T	nonsynonymous
	ARID1A	P13	Target	NM_006015	G	A	p.V1982I	nonsynonymous
	ARID1A	P31	WES	NM_006015	-	A	p.P421fs	frameshift
	ARID1A	P40	WES	NM_006015	C	T	p.R1335X	stopgain
	ARID1A	P42	Target	NM_006015	T	G	p.Y1012X	stopgain
	ARID1A	P43	Target	NM_006015	C	T	p.A1872V	nonsynonymous
	ARID2	P36	WES	NM_001347839	C	T	p.P536S	nonsynonymous
	CHD8	P14	WES	NM_001170629	G	A	p.S2069L	nonsynonymous
Tumor	*TP53*	P3	Target	NM_000546	C	T	p.R273C	nonsynonymous
suppressor	*TP53*	P26	WES	NM_000546	C	T	p.V31I	nonsynonymous

	TP53	P31	WES	NM_001126118	-	G	p.P8fs	frameshift
	TP53	P37	WES	NM_001126115	A	C	p.V41G	nonsynonymous
	ATM	P9	Target	NM_000051	T	C	p.I1688T	nonsynonymous
	ATM	P11	WES	NM_000051	C	T	p.P2974L	nonsynonymous
	ATM	P32	WES	NM_000051	G	A	p.D520N	nonsynonymous
	MGA	P10	WES	NM_001164273	G	A	p.V1699I	nonsynonymous
	MGA	P11	WES	NM_001080541	A	G	p.T338A	nonsynonymous
	NF1	P33	WES	NM_001128147	G	C	p.R574S	nonsynonymous
JAK-STAT	*SOCS1*	P31	WES	NM_003745	G	A	p.Q131X	stopgain
pathway	*SOCS1*	P34	Target	NM_003745	506_529del	506_529del	p.169_177del	frameshift
	SOCS1	P35	Target	NM_003745	106_122del	106_122del	p.P36fs	frameshift
	JAK3	P33	WES	NM_000215	C	T	p.M511I	nonsynonymous
	STAT3	P32	WES	NM_003150	C	T	p.G421R	nonsynonymous
Transcriptional	*ASXL3*	P8	Target	NM_030632	C	G	p.P1457R	nonsynonymous
regulation	*PRDM1*	P26	WES	NM_001198	G	A	p.G11D	nonsynonymous
Others	*RHOA*	P5	WES	NM_001313943	C	A	p.G17V	nonsynonymous
	RHOA	P6	WES	NM_001313943	C	A	p.G17V	nonsynonymous
	RHOA	P26	WES	NM_001313943	G	T	p.G17V	nonsynonymous
	RHOA	P27	WES	NM_001313943	C	A	p.G17V	nonsynonymous
	RHOA	P28	WES	NM_001313943	C	A	p.G17V	nonsynonymous
	RHOA	P29	Target	NM_001664	G	T	p.G17V	nonsynonymous
	RHOA	P30	WES	NM_001313943	C	A	p.G17V	nonsynonymous
	NOTCH1	P7	WES	NM_017617	C	T	p.R2263Q	nonsynonymous

Table S3. Univariable analysis of factors associated with progression-free survival and overall survival

		Progression-free survival			Overall survival		
	N	Median (95% CI)	HR (95% CI)	*p*-value	Median (95% CI)	HR (95% CI)	*p*-value
Age							
≤ 60 years	61	13.8 (7.4-20.2)			25.2 (15.5-34.9)		
> 60 years	41	11.6 (5.8-17.4)	1.30 (0.81-2.09)	0.279	23.8 (14.8-32.8)	1.56 (0.92-2.65)	0.098
Gender							
Male	66	13.8 (6.9-20.7)			24.3 (15.1-33.6)		
Female	36	7.3 (0.2-14.5)	1.08 (0.66-1.78)	0.750	22.3 (9.2-35.3)	1.12 (0.64-1.95)	0.701
Pathology type							
PTCL*	52	12.4 (7.8-16.9)		**0.005**	21.9 (15.7-28.1)		**0.029**
AITL	34	7.5 (3.4-11.6)	1.16 (0.71-1.90)	0.573	23.3 (15.8-30.7)	1.15 (0.66-2.00)	0.615
ALCL	16	Not reached	0.23 (0.80-0.65)	**0.003**	Not reached	0.25 (0.08-0.83)	**0.016**
IPI							
0-1	30	Not reached		**0.004**	Not reached		**0.000**
2	28	14.5 (1.7-27.3)	2.08 (1.02-4.23)	**0.034**	25.2 (19.8-30.7)	2.58 (1.09-6.12)	**0.019**
3	28	11.6 (4.9-18.3)	2.28 (1.15-4.51)	**0.021**	18.5 (14.4-22.7)	3.54 (1.54-8.11)	**0.002**
4-5	16	3.5 (0.0-13.1)	3.59 (1.65-7.83)	**0.003**	8.8 (0.0-18.6)	6.67 (2.69-16.57)	**0.000**
Treatment							
CEOP/IVE/GDP	51	15.4 (9.4-21.5)			24.3 (17.1-31.6)		
CEOP	51	9.2 (4.2-14.2)	1.36 (0.85-2.19)	0.199	23.3 (9.6-37.0)	1.44 (0.84-2.47)	0.178
EBV-DNA (N=87)							
Undetectable	65	15.1 (11.0-19.3)			29.4 (20.1-38.7)		
Detectable	22	5.3 (2.1-8.6)	1.76 (1.00-3.10)	**0.046**	8.8 (2.3-15.3)	2.43 (1.30-4.53)	**0.004**
Gene Mutation (N=62)							
KMT2D positive	14	6.9 (6.5-7.3)	2.00 (0.99-4.01)	**0.047**	28.1 (0.0-64.1)	0.94 (0.39-2.29)	0.890
KMT2A	2	4.6 (NA)	1.33 (0.32-5.54)	0.690	6.1 (NA)	2.29 (0.54-9.66)	0.248
EP300	5	25.2 (NA)	0.51 (0.12-2.11)	0.343	25.2 (NA)	0.36 (0.05-2.67)	0.298
CREBBP	3	4.3 (3.0-5.6)	4.89 (1.41-16.89)	**0.005**	4.3 (3.1-5.5)	7.27 (2.04-25.84)	**<0.001**
TET2	14	8.0 (0.0-22.2)	0.90 (0.45-1.82)	0.768	24.3 (8.2-40.4)	1.22 (0.55-2.70)	0.631
DNMT3A	7	25.2 (8.5-41.9)	0.49 (0.18-1.38)	0.167	25.2 (18.4-32.0)	1.13 (0.40-3.23)	0.821
IDH2	3	3.5 (3.3-3.7)	8.21 (2.21-30.42)	**<0.001**	8.9 (1.2-16.4)	7.29 (1.99-26.74)	**<0.001**
ARID1B	9	5.0 (3.8-6.2)	2.31 (1.07-5.01)	**0.028**	9.3 (8.0-10.6)	2.91 (1.30-6.51)	**0.006**
ARID1A	6	10.6 (3.9-17.3)	1.26 (0.49-3.21)	0.626	29.4 (17.4-41.4)	0.69 (0.21-2.26)	0.436
TP53	4	7.4 (NA)	0.54 (0.13-2.25)	0.392	Not reached	0.44 (0.06-3.19)	0.398
ATM	3	8.0 (6.7-9.3)	1.96 (0.59-6.51)	0.258	15.7 (7.6-23.8)	2.70 (0.80-9.06)	0.094
MGA	2	6.9 (NA)	2.03 (0.48-8.56)	0.322	10.67 (NA)	4.16 (0.94-18.49)	0.061
SOCS1	3	7.4 (5.6-9.2)	1.51 (0.47-4.90)	0.487	15.6 (4.6-65.7)	0.74 (0.18-3.09)	0.672
RHOA	7	15.9 (0.0-35.1)	0.96 (0.41-2.32)	0.957	24.3 (0.8-47.8)	1.32 (0.46-3.78)	0.604

* PTCL subtype includes PTCL, NOS and other types.

NA= not available. CEOP = cyclophosphamide, epirubicin, vincristine, and prednisone. IVE = ifosmide, epirubicin, and etoposide. GDP = gemcitabine, cisplatin, and dexamethasone. LDH = lactate dehydrogenase. IPI = International Prognostic Index.

Table S4. Multivariate analysis of factors associated with progression-free survival and overall survival

	Progression-free survival				Overall survival		
	N	HR	95% CI	*p*-value	HR	95% CI	*p*-value
Pathology type							
PTCL*	37	1.0		(0.410)	1.0		(0.551)
AITL	18	0.81	0.29-2.21	0.673	0.80	0.27-2.42	0.564
ALCL	7	0.35	0.07-1.65	0.183	0.31	0.12-0.76	0.062
IPI							
0-1	16	1.0		(0.336)	1.0		(0.120)
2	20	0.68	0.26-1.78	0.437	1.50	0.39-5.84	0.558
3	17	1.37	0.52-3.61	0.530	2.80	0.79-9.90	0.111
4-5	9	1.71	0.60-4.89	0.319	4.48	1.20-16.76	0.026
EBV-DNA							
Undetectable	45						
Detectable	14	1.34	0.53-3.40	0.544	1.50	0.54-4.16	0.435
Gene Mutation							
KMT2D positive	14	3.12	1.50-6.50	**0.002**	/	/	/
ARID1B	9	1.43	0.39-5.22	0.592	3.16	0.82-12.23	0.096
CREBBP	3	11.13	2.91-42.51	**<0.001**	10.27	2.75-38.35	**0.001**
IDH2	3	19.61	4.66-82.44	**<0.001**	10.44	2.69-40.61	**0.001**

* PTCL subtype includes PTCL, NOS and other types.

CEOP = cyclophosphamide, epirubicin, vincristine, and prednisone. IVE = ifosmide, epirubicin, and etoposide. GDP = gemcitabine, cisplatin, and dexamethasone. LDH = lactate dehydrogenase. IPI = International Prognostic Index.

Table S5. Progression-free survival and overall survival between two study groups in terms of IPI, NCCN-IPI and PIT

	N	Median progression-free survival			Median overall survival		
		CEOP/IVE/GDP	CEOP	*p*-value	CEOP/IVE/GDP	CEOP	*p*-value
IPI							
0-1	30	Not reached	Not reached	0.713	Not reached	Not reached	0.565
2	28	16.8 (2.8-30.8)	14.5 (1.0-28.0)	0.917	25.2 (14.4-35.0)	30.0 (20.3-39.6)	0.616
3	28	15.1 (10.7-19.7)	7.3 (1.1-13.5)	0.210	20.8 (12.3-29.2)	12.9 (5.1-20.7)	0.328
4-5	16	15.4 (1.3-29.6)	2.6 (1.0-4.2)	0.089	20.1 (15.4-24.8)	4.6 (0.0-11.3)	**0.012**
NCCN-IPI							
0-1	11	Not reached	Not reached	0.553	Not reached	Not reached	0.987
2-3	44	13.8 (2.0-25.6)	15.9 (0.0-32.0)	0.229	22.3 (15.6-28.9)	41.7 (26.1-57.3)	0.141
4-6*	47	16.8 (10.1-23.5)	5.8 (3.1-8.5)	**0.024**	23.8 (18.6-29.0)	9.4 (5.8-12.9)	**0.028**
PIT							
0	24	Not reached	Not reached	0.881	Not reached	Not reached	0.515
1	34	15.1 (1.7-28.6)	9.2 (0.0-30.9)	0.698	24.3 (13.2-35.5)	27.9 (17.0-38.8)	0.880
2	26	16.8 (0.7-32.9)	7.3 (0.0-16.3)	0.465	23.8 (20.7-26.9)	11.1 (7.1-15.1)	0.401
3-4	18	15.4 (7.7-23.2)	2.6 (0.3-4.9)	**0.026**	20.1 (15.3-24.9)	4.6 (0.0-13.8)	**0.021**

*Including NCCN-IPI of 6 because of only 1 patient in this category

IPI=International prognostic index, NCCN-IPI= National Comprehensive Cancer Network- International prognostic index, PIT= Prognostic Index for T cell lymphoma.
